# m6A regulator-mediated methylation modification patterns and tumor microenvironment infiltration characterization in hepatocellular carcinoma

**DOI:** 10.18632/aging.203456

**Published:** 2021-08-30

**Authors:** Xiongpei Huang, Zecheng Qiu, Liusheng Li, Bin Chen, Peiyuan Huang

**Affiliations:** 1 Department of Pharmacy, Gaozhou People’s Hospital, Gaozhou 525200, China; 2 Department of Hepatological Surgery, Maoming People’s Hospital, Maoming 525000, China

**Keywords:** immunotherapy, m6A, methylation, tumor microenvironment, hepatocellular carcinoma

## Abstract

Background: There is increasing evidence of the epigenetic regulation of the immune response in cancer. However, the specific functions and mechanisms of RNA N6-methyladenosine (m6A) modification in the cell infiltration in the hepatocellular carcinoma (HCC) tumor microenvironment (TME) is unknown.

Methods: We systematically analyzed the m6A-modification patterns of 371 HCC samples based on 23 m6A regulators, and determined their correlation with TME cell-infiltrating characteristics. Principal-component analysis algorithms was used to calculate the m6Ascore and clarify the m6A-modification patterns of individual tumors.

Results: Three different m6A-modification patterns were identified in HCC, wherein the m6Acluster B and m6Acluster A had the best and worst prognosis, respectively. These three patterns had different TME cell infiltration characteristics and biological behavior. An m6A-scoring signature was constructed to evaluate the m6A-modification patterns within individual tumors. A low m6Ascore was associated with a low overall survival and high clinical stage. Moreover, the m6A-scoring signature was characterized by distinct immunotherapeutic landscapes; a high m6A score indicated a higher immune checkpoint inhibitor score in the anti-PD-1 treatment alone, anti-CTLA-4 treatment alone, or combined anti-CTLA-4/PD-1 treatment cohorts, which reflected significant treatment and clinical benefits.

Conclusions: Our study highlights the significant role of the m6A modification in the HCC TME. A scoring signature to clarify the individual m6A-modification pattern would help us understand the HCC TME infiltration characterization and, thus, would guide the selection of more effective immunotherapeutic strategies.

## INTRODUCTION

Hepatocellular carcinoma (HCC) is a highly prevalent malignancy that usually emerges from chronic liver disease [1]. Worldwide, in 2018, HCC was the fifth and ninth commonest malignancy in men and women, respectively, and the second commonest cause of cancer-related deaths, with an estimated 841,100 new HCC cases and 781,600 HCC-related deaths [2, 3]. With the technological advances in diagnostic methods in the past decade, the incidence of HCC has continued to increase [4]. Despite the use of multidisciplinary synthetic therapy after hepatectomy, HCC cells can escape these cancer therapeutics, leading to cancer recurrence, metastasis, and, eventually, death [5]. HCC has a poor prognosis, with a median survival of approximately 12 months [5]. These data demonstrate the need of a reliable signature for tumor classification and prognosis to facilitate the planning of the therapeutic strategy in patients with HCC.

The N6-methyladenosine (m6A) RNA methylation is the most significant and abundant form of RNA modification in eukaryotic cells and constitutes 0.3% of all the adenosine residues [6, 7]. Modulated by three different m^6^A regulatory factors (“writers” for methyltransferases, “erasers” for demethylases, and “readers” for binding proteins), m^6^A modification is a dynamic and reversible process that dynamically regulates RNA translation, degradation, and nuclear output in human cells [8]. Thus far, 23 regulators have been identified, including the methyltransferases METTL3, METTL14, METTL16, WTAP, VIRMA, ZC3H13, RBM15, and RBM15B; the binding proteins YTHDC1, YTHDC2, YTHDF1, YTHDF2, YTHDF3, HNRNPC, FMR1, LRPPRC, HNRNPA2B1, IGFBP1, IGFBP2, IGFBP3, and RBMX; and the two demethylases FTO and ALKBH5 [9]. The participation of m6A regulators in various cancer biological processes, including proliferation, invasion, and metastasis, has been increasingly proven [10]. Moreover, the pattern of m6A regulator expression is associated not only with the immune microenvironment but also predicts the therapeutic efficacy and prognosis based on the tumor [11].

With the rapid development of gene sequencing technology and the establishment of databases, including the Cancer Genome Atlas (TCGA; https://cancergenome.nih.gov/), bioinformatics analysis based on database mining has emerged as one of the most promising options for translational research in cancer. In this study, we aimed to comprehensively explore the expression and prognostic implications of RNA m6A modification in HCC. Based on the expression pattern of m6A regulators, we performed consensus clustering using principal components analysis (PCA), survival analysis, and single-sample gene-set analysis (ssGSEA). Furthermore, we constructed an m6A-scoring signature and explored its association with the immune microenvironment and immunotherapy.

## MATERIALS AND METHODS

### Collection and preprocessing of the HCC dataset

Gene expression data for HCC samples based on clinical annotations were obtained from the public TCGA dataset (https://cancergenome.nih.gov/), and RNA sequencing data (FPKM format) for HCC were converted to the transcripts per kilobase million (TPM) format. The clinical annotations that were collected for HCC included survival time, survival status, age, sex, stage, grade and TNM staging. HCC genomic mutation data (including somatic mutations and copy number variants (CNV) were obtained, and the R packages “maftools” and “Rcircos” were used for the detection of somatic mutations and copy number visualization. RNA-seq data and clinical survival information for an additional 115 HCC samples were obtained from the GSE76427 (https://www.ncbi.nlm.nih.gov/geo/) as the testing dataset.

### Consensus clustering of m6A regulators

We searched the literature for reports related to m6A methylation regulators, and finally identified 23 m6A-regulated regulators, including 8 writers (METTL3, METTL14, METTL16, RBM15, RBM15B, WTAP, ZC3H13, VIRMA [KIAA1429]), 2 erasers (FTO and ALKBH5), and 13 readers (YTHDC1, YTHDC2, YTHDF1, YTHDF2, YTHDF3, HNRNPC, FMR1, LRPPRC, HNRNPA, and ALKBH5) for analysis [9].

First, a univariate Cox model was used to calculate the association between the expression level of each m6A regulator and the overall survival (OS) of patients; the m6A regulator was considered to be associated with the prognosis when *p* < 0.05. Then, consistent clustering was performed using the R package ”Consensus Cluster Plus” and 1000 cycles were undertaken to ensure the stability of the classification; the number of cluster k-values were increased from 2 to 9. The k-values with better clustering stability were selected according to the clustering effect [12], and heatmaps corresponding to consistent clustering were generated by the pheatmap R package.

### Gene set variation analysis (GSVA) and estimation of immune cell infiltration by single-sample gene set enrichment analysis (ssGSEA)

GSVA comprised a non-parametric, unsupervised analytical method that is mainly used to evaluate the results of transcriptome gene set enrichment [13]. First, the gene set “c2.cp.kegg.v7.2.symbols.gmt” was downloaded from the MSigDB database (https://www.gsea-msigdb.org) for subsequent enrichment analysis. We performed GSVA enrichment analysis of m6A typing by using the R package “GSVA” to investigate the variation of biological processes between the m6A types. An adjusted *p* < 0.05 was considered statistically significant. To determine the extent of immune cell infiltration in the m6A typing, we used the validated ssGSEA, wherein the extent of each immune cell infiltration in each sample was expressed by the enrichment score calculated from the ssGSEA analysis. The analysis of a total of 23 sets of gene markers that were used to identify infiltrating immune cells was undertaken with the R package “GSVA,” based on Charoentong's study [14].

### Screening and consensus clustering of differentially expressed genes (DEGs) for the determination of m6A modification patterns

Based on the previous consensus clustering algorithm, patients were classified into three different m6A modification patterns, and DEGs among HCC patients with different m6A modification patterns were screened using the R package “limma.” DEGs that were considered significant based on an adjusted *p* < 0.0001 were identified. Similarly, the univariate Cox model was used to screen out survival-related DEGs for the three distinct m6A modification patterns, and the R package “Consensus Cluster Plus” was used for consistent clustering. Finally, the survival of different genotypes and the results of the analysis of the differences in the expression of m6A regulators were compared.

### Development of an m6A-scoring signature

In conjunction with previous studies, an m6A scoring system was developed, and genes with significant prognosis were selected by univariate Cox regression analysis. PCA analysis was conducted to extract principal components, and the principal components 1 and 2 were selected as feature scores. This approach focused on the scores on the set of gene blocks with the greatest correlation (or inverse correlation) whereas eliminating, to the extent possible, the contribution of genes that do not track other members. Then, based on previous research [15, 16], the m6A score was calculated using the formula:

$$m6A-scoring=\sum \left({\text{PC1}}_{i}+\text{PC}2_{i}\right)$$

where it is the finalized expression of the m6A phenotype-associated genes.

To determine the feasibility and reliability of the m6A-scoring signature, we used the testing set of GSE76427 samples (n=115) in the same methods as described above.

### Correlation of the m6A-scoring signature with genomic mutations, clinical information, and immunity

The samples were divided into high- and low-m6A score groups according to the m6A score, and the differences between the two groups in terms of mutation, tumor mutation load, and clinical annotation were ascertained. Furthermore, with regard to immunity, ssGSEA was used to quantify the subset of tumor-infiltrating immune cells between the two m6A-score groups and to assess their immunological differences. Moreover, intergroup differences in potential immune checkpoints, such as PD-L1, PD-1, CTLA4, LAG3, TIGIT, and TIM-3, were analyzed using the Wilcoxon test.

Finally, for immunotherapy, the immune checkpoint inhibitor (ICI) immunophenoscore (IPS) file was downloaded from The Cancer Immunome Database; the IPS is a good predictor of CTLA-4 and PD-1 responsiveness, and predicts the intergroup differences in response to immunotherapy using CTLA-4 and PD-1 blockers [14, 17, 18].

### Statistical analysis

For the comparison of data between the two groups, we used the *t*-test for variables conforming to a normal distribution and the non-parametric test (Wilcoxon rank sum test) for variables with non-normal distribution. For the comparison of data from more than two groups, one-way ANOVA and the Kruskal–Wallis test were used as parametric and non-parametric methods, respectively. The best cutoff score between the two groups of high- and low-m6A score was derived by the surv-cutpoint function. The Kaplan–Meier method and log-rank test were used for the survival analysis. A *p*-value < 0.05 defined statistical significance in this study. All statistical analyses were performed using R Studio (3.6.1).

## RESULTS

### Genetic variation profile of the m6A regulators in HCC

**Figure 1:**
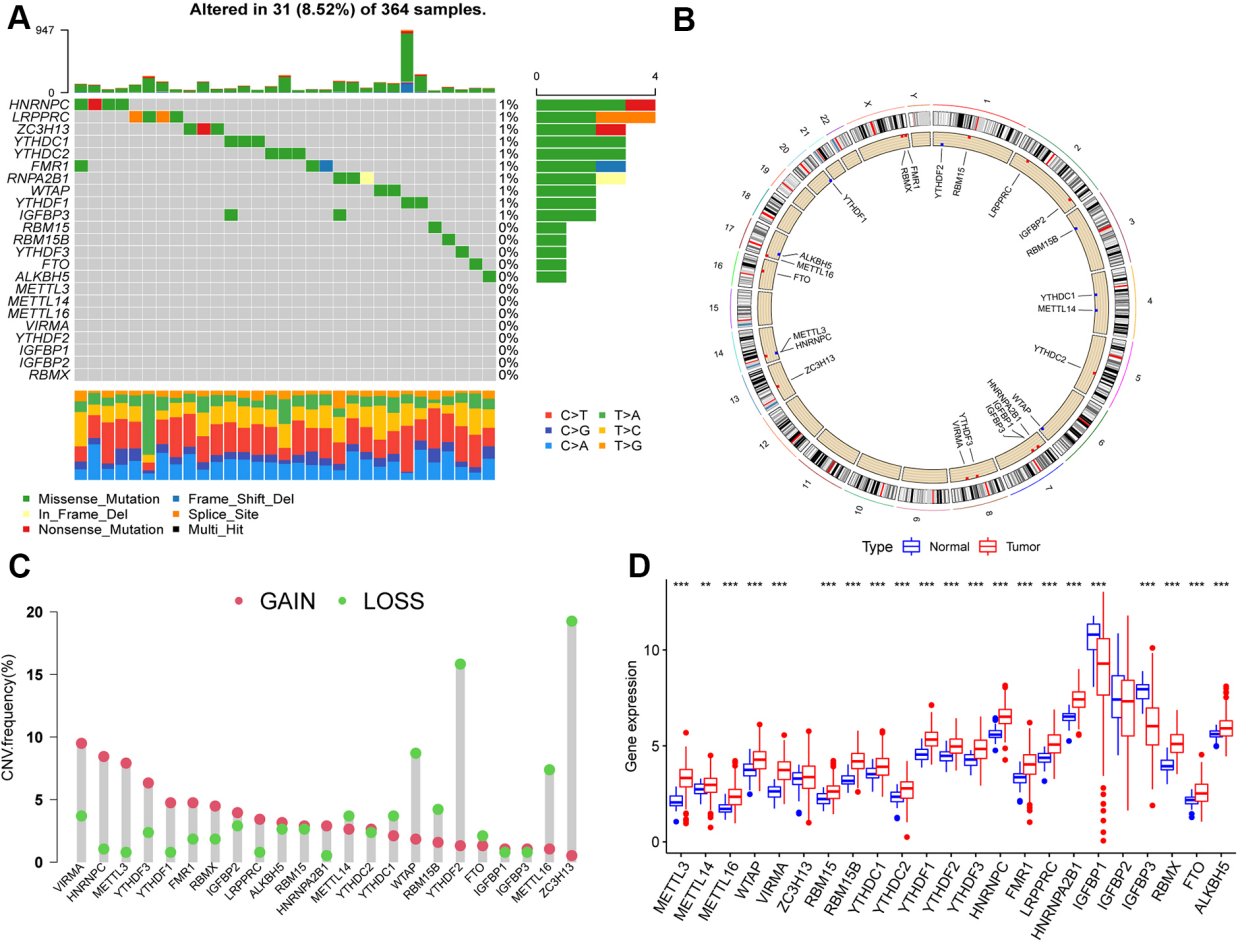
**Landscape of genetic variation of m6A regulators in hepatocellular carcinoma (HCC).** (**A**) Genetic alteration on a query of m6A regulators. (**B**) The position of the CNV alteration of the m6A regulators on 23 chromosomes from the TCGA-LIHC cohort. (**C**) The CNV variation frequency of m6A regulators. Red dots represent CNV amplification, while green dots represent CNV deletion. Compared to the other m6A regulators, ZC3H13, YTHDF2 and WTAP had a higher frequency of CNV deletion, while VIRMA, HNRNPC and METTL3 had a higher frequency of CNV amplification. (**D**) The gene expression levels of 23 m6A regulators in HCC (*, P < 0.05; **, P < 0.01; ***, P < 0.001; ns, no significant). Compared with the normal tissue, the expression of METTL3, METTL14, METTL16, WTAP, VIRMA, RBM15, RBM15B, YTHDC1, YTHDC2, YTHDF1, YTHDF2, YTHDF3, HNRNPC, FMR1, LRPPRC, HNRNPA2B1, RBMX, FTO, and ALKBH5 were upregulated, IGFBP1and IGFBP3 were downregulated in HCC.

We first summarized the somatic mutations and copy numbers of 23 m6A regulators in HCC. Only 31 of the 364 samples (8.52%) experienced genetic alterations in the m6A regulator, and the overall mutation frequencies were all low (≤1%; Figure 1A). Figure 1B shows the location of CNV alterations on the chromosome for m6A regulators, and that CNV changes were prevalent in these regulators, with higher frequencies of CNV deletions in ZC3H13, YTHDF2, and WTAP and, conversely, higher probabilities of CNV amplification in VIRMA, HNRNPC, and METTL3 (Figure 1C).

Furthermore, we found that METTL3, METTL14, METTL16, WTAP, VIRMA, RBM15, RBM15B, YTHDC1, YTHDC2, YTHDF1, YTHDF2, YTHDF3, HNRNPC, FMR1, LRPPRC, HNRNPA2B1, RBMX, FTO, and ALKBH5 were significantly upregulated in HCC tissues compared to their expression in normal tissues, whereas IGFBP1 and IGFBP3 were significantly downregulated (*p* < 0.05; Figure 1D).

### Immune infiltration and biological functions with m6A methylation-modification patterns

**Figure 2:**
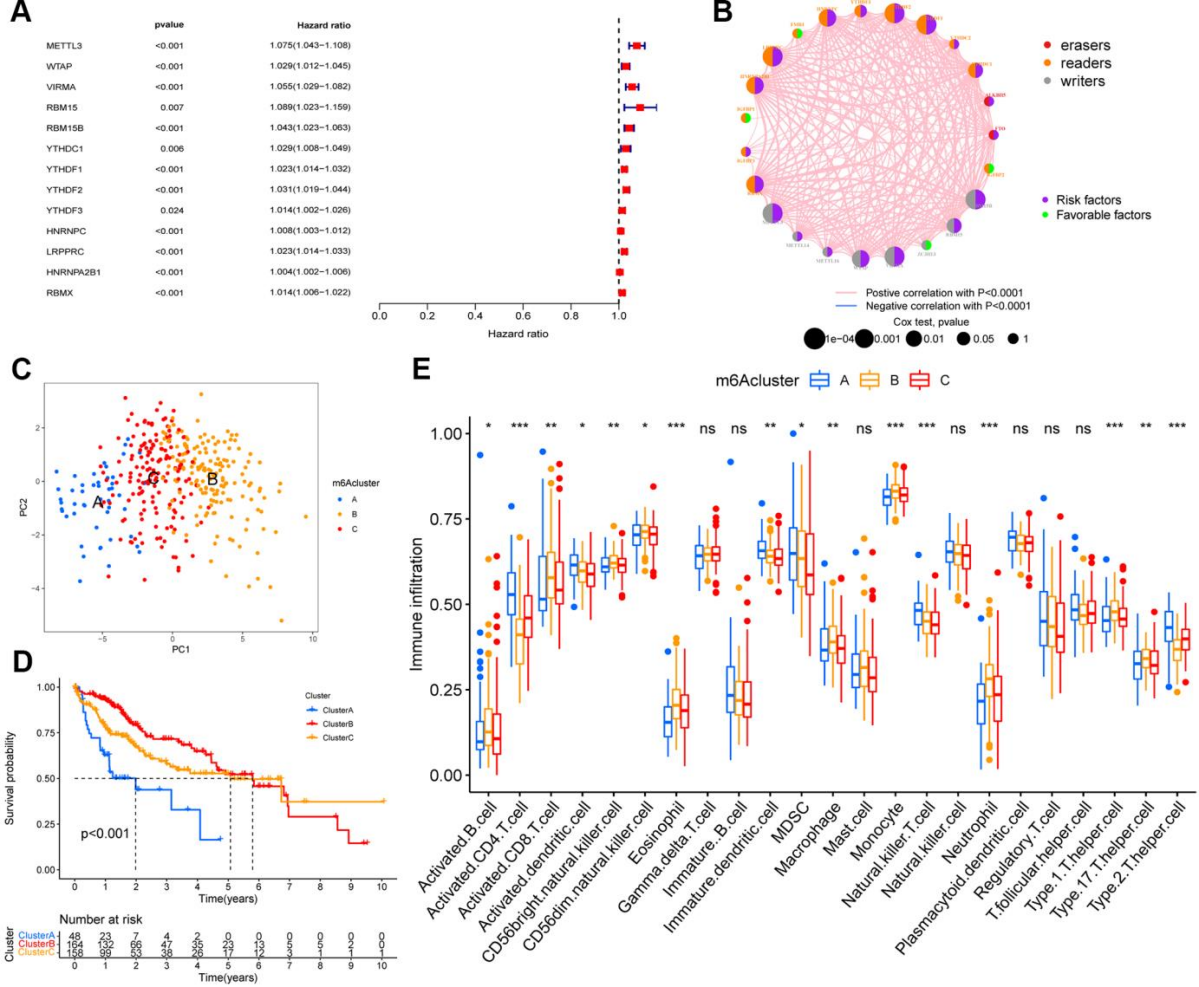
**m6A methylation modification pattern and relevant immune infiltration.** (**A**) Prognosis of 23 m6A regulators was analyzed using univariate Cox regression models. METTL3, WTAP, VIRMA, RBM15, RBM15B, YTHDC1, YTHDF1, YTHDF2, YTHDF3, HNRNPC, LRPPRC, HNRNPA2B1, RBMX were risk factors (Hazard ratio >1). (**B**) Interactions between m6A regulators in hepatocellular carcinoma. The size of the circles represents the effect of each modulator on prognosis, with larger circles having a greater effect on prognosis (p-values from 1 to 0.0001). Circles with purple and green colors indicate prognostic risk and protective factors respectively. The line connecting the m6A regulators represents the correlation between the m6A regulators, with negative correlations marked in blue and positive correlations marked in pink. (**C**) Principal component analysis (PCA) analysis of m6A methylation modification pattern. (**D**) The overall survival of m6A methylation modification pattern using Kaplan–Meier curves. (**E**) Differences in immune cell infiltration of m6A methylation modification pattern (*, P < 0.05; **, P < 0.01; ***, P < 0.001; ns, no significant; Kruskal–Wallis test).

We used univariate Cox regression analysis to screen for m6A regulators associated with prognosis in HCC (Figure 2A), and constructed interaction network plots between m6A regulators to demonstrate their interactions (Figure 2B). In order to further clarify the clinical or biological value of these m6A regulators, we performed consensus clustering and divided the TCGA-HCC cohort samples into subgroups based on the expression of 23 m6A regulators. Thus, k=3 was found to confer optimal clustering stability from k=2 to k=9, and TCGA LIHC patients were classified based on three different m6A modification patterns (Supplementary Figure 1A–1C), as m6Acluster A (n=48), m6Acluster B (n=164), and m6Acluster C (n=158); among these, the m6Acluster B had the best prognosis and the m6Acluster A had the worst prognosis (Figure 2D). These subgroups with different clinicopathological characteristics are shown in Supplementary Figure 1D. Furthermore, we noted differences in immune cell infiltration between the different m6A modification patterns, including the infiltration pattern of activated CD4 T cell, activated CD8 T cell, activated dendritic cell, CD56bright natural killer cell, CD56dim natural killer cell, eosinophil, immature dendritic cell, MDSC, macrophage, monocyte, natural killer T cell, neutrophil, Type 1 T helper cell, Type 17 T helper cell, and Type 2 T helper cell. Compared with the other two m6A modification modes, the m6Acluster B had a higher abundance of immune-infiltrating cells in the activated B cell, activated CD8 T cell, CD56bright natural killer cell, CD56dim natural killer cell, eosinophil, macrophage, monocyte, neutrophil, Type 1 T helper cell, and Type 17 T helper cell (Figure 2E). We performed GSVA enrichment analysis in order to explore the biological behaviors among the m6A modification patterns. The m6Acluster A was significantly enriched in cell cycle and RNA degradation, whereas the m6Acluster B and m6Acluster C showed PPAR signaling pathway and enrichment pathways related to tumor metabolism, including the metabolism of xenobiotics by cytochrome P450, drug metabolism cytochrome P450, metabolism of alpha-linolenic acid, linoleic acid, and arachidonic acid (Supplementary Figure 2).

### Identification of the m6A modification genomic phenotypes

**Figure 3:**
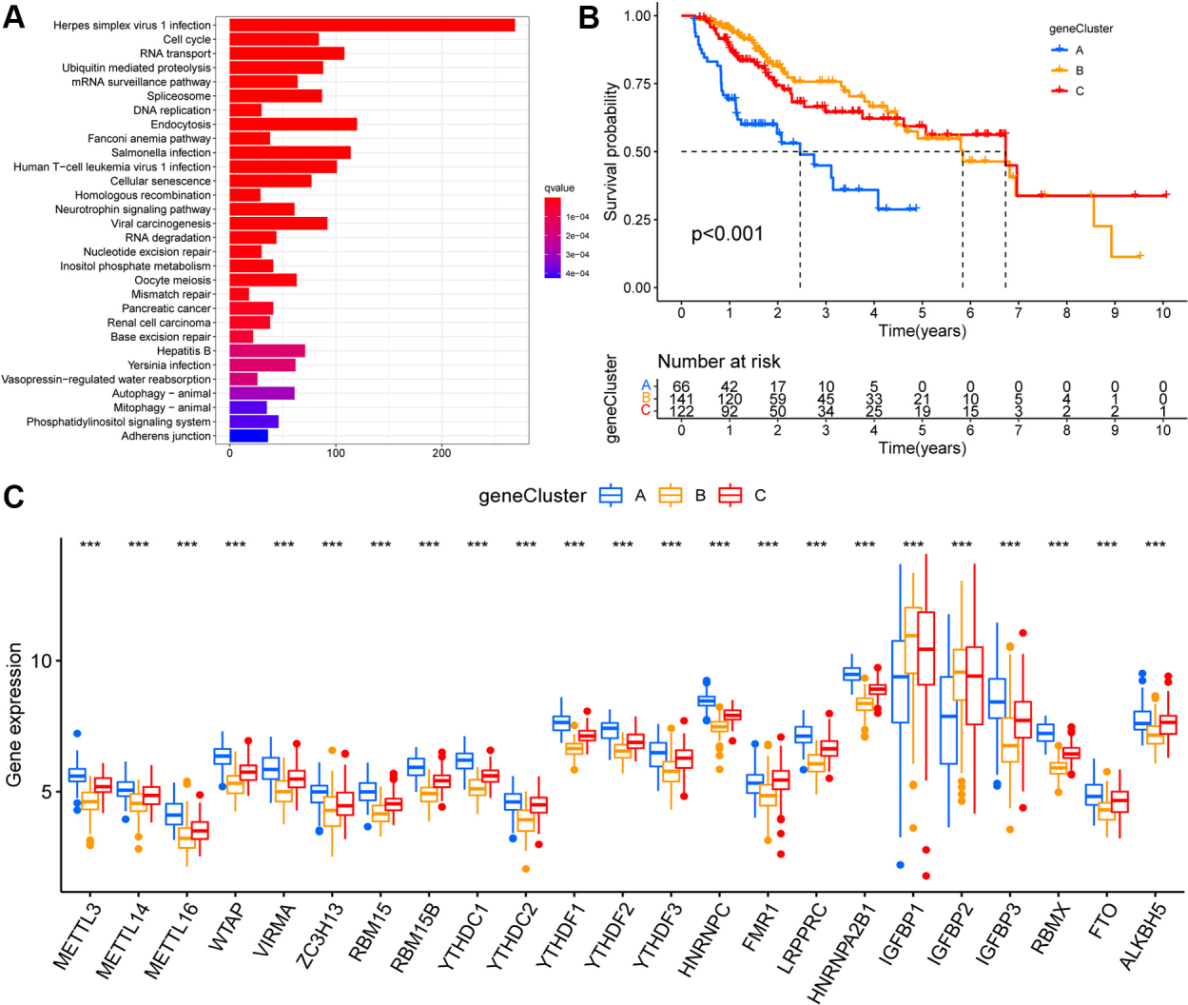
**Identification of m6A modification genomic phenotypes in hepatocellular carcinoma.** (**A**) Biological pathway of differentially expressed genes (DEGs) for the m6A modification patterns. (**B**) The overall survival of m6A modification genomic phenotypes using Kaplan–Meier curves. (**C**) The gene expression levels of 23 m6A regulators in three m6A modification genomic phenotypes (*, P < 0.05; **, P < 0.01; ***, P < 0.001; ns, no significant; Kruskal–Wallis test).

Based on the above-described three m6A modification patterns in HCC, we further analyzed potential m6A-related transcriptional expression changes between the modification patterns, and identified 5372 overlapping m6A phenotype-related DEGs (Supplementary Figure 3A). Moreover, KEGG enrichment analysis was performed (Figure 3A). These DEGs were mainly enriched in in hepatitis virus infection and the cell-cycle pathways.

Based on these DEGs, we performed unsupervised cluster analysis and identified three m6A modified genomic phenotypes (Supplementary Figure 3B–3D), named geneCluster A, geneCluster B, and geneCluster C, wherein geneCluster B showed a significant survival advantage, followed by geneCluster C, and geneCluster A showed the lowest prognostic advantage (Figure 3B). These subgroups had different clinicopathological characteristics (Supplementary Figure 3E). Between these three m6A-modified genomic phenotypes, we observed significant differences in the expression of m6A regulators. Compared to geneCluster A, 21 m6A regulators (except IGFBP1 and IGFBP2) were expressed at higher and lower levels in geneCluster C and geneCluster B, respectively (Figure 3C).

### Construction of the m6A-scoring signature

**Figure 4:**
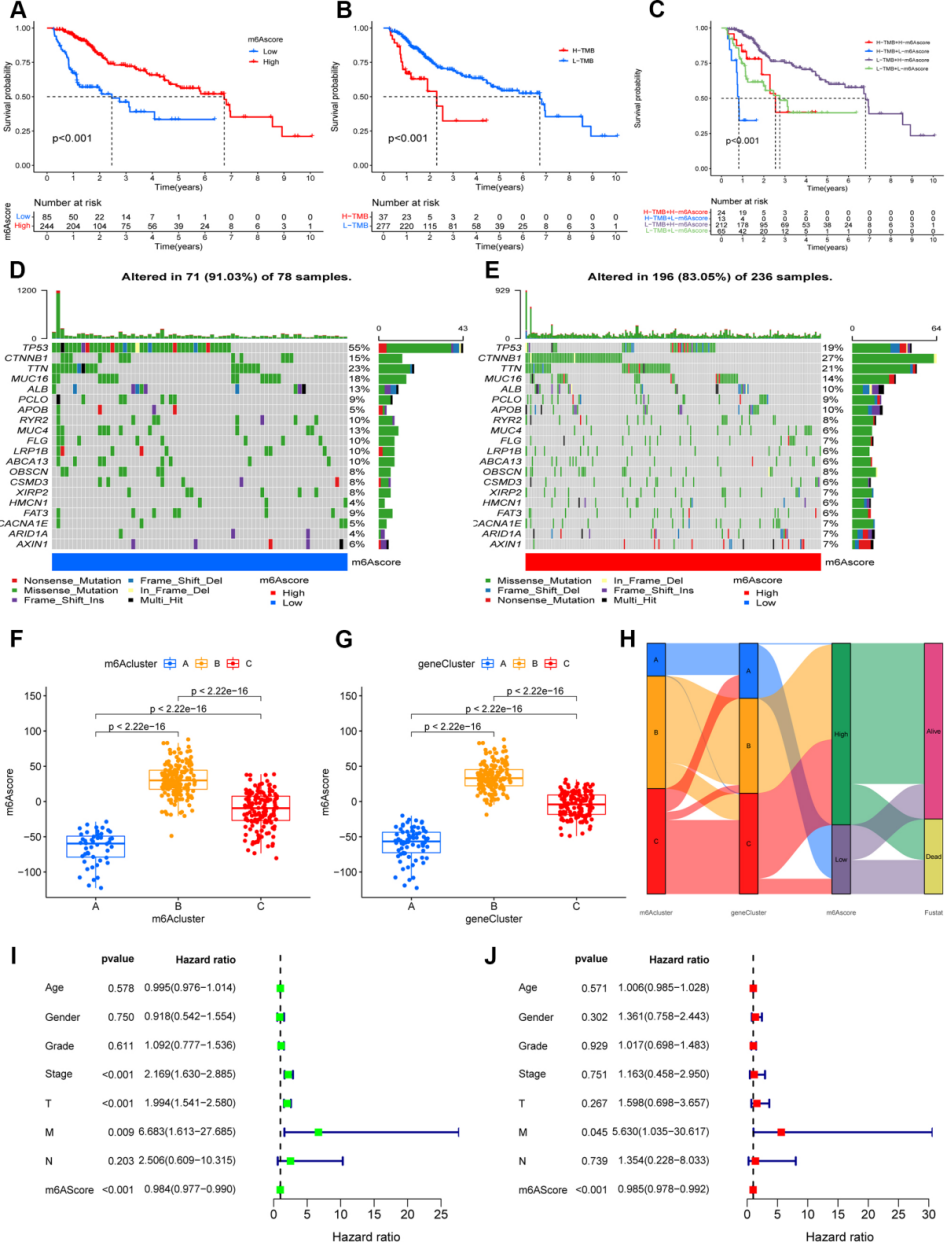
**Construction the m6A-scoring signature.** (**A**) The overall survival of m6A-scoring signature using Kaplan–Meier in Log-rank test. (**B**) The overall survival of low and high tumor mutation burden (TMB) score groups using Kaplan–Meier in Log-rank test. (**C**) The overall survival of the patients stratified by both the m6A-scoring signature and TMB using Kaplan–Meier curves. Mutation spectrum of the low (**D**) and high (**E**) m6A score groups. (**F**) Differences in m6A score group among three m6A methylation modification patterns (m6Acluster) (P < 0.001, Kruskal–Wallis test). (**G**) Differences in m6A score group among three m6A modification genomic phenotypes (geneCluster) (P < 0.001, Kruskal–Wallis test). (**H**) Alluvial diagram showing the changes of m6A methylation modification patterns (m6Acluster), m6A modification genomic phenotypes (geneCluster), m6Ascore, and survival status (Fustat). (**I**) Univariate COX analysis for the m6A-scoring signature. (**J**) Multivariate COX analysis for the m6A-scoring signature.

The analysis described above is largely based on a large population, and these methods cannot be applied to accurately predict the pattern of m6A methylation modifications in individual HCC patients. In view of the heterogeneity and complexity of each patient, we constructed a scoring system to judge the pattern of m6A modification in each HCC patient, and used an alluvial diagram to analyze the attribute changes of individual patients (Figure 4H). Survival analysis showed that patients with high m6A scores showed a significant survival benefit (*p* < 0.001; Figure 4A), whereas patients with low TMB scores showed a significant survival benefit (*p* < 0.001; Figure 4B), and patients with high m6Sig scores combined with low TMB scores also showed a significant survival benefit (*p* < 0.001; Figure 4C). In the testing set, patients in the low-m6A score group survived for a shorter period compared to those in the high-m6A score group (Supplementary Figure 4). Moreover, we analyzed tumor somatic mutations in the high and low m6Sig score subgroups separately, and found a higher somatic mutation rate in the low-m6A score group for TP53 (55% vs. 19%) and a lower somatic mutation rate for CTNNB1 (15% vs. 27%) (Figure 4D, 4E). The Kruskal–Wallis test revealed significant differences in m6A scores between m6A methylation modification patterns (m6Acluster), with m6Acluster B and m6Acluster A having the highest and lowest median scores, respectively (Figure 4F). Furthermore, there were significant differences in the m6A scores between the m6A modified genomic phenotypes (geneCluster), with geneCluster B and geneCluster A having the highest and lowest median scores, respectively, compared to the other subgroups (Figure 4G).

In the clinical correlation analysis, we found that younger, surviving, G1–2, Stage I–II, T1–2, and N1 patients were significantly associated with a lower m6A score (*p* < 0.05; Supplementary Figure 5). Univariate and multifactorial Cox regression model analyses including the patient’s age, sex, stage, grade, and TNM staging confirmed that the m6A-scoring signature was an independent prognostic factor for OS in HCC patients (*p* < 0.001; Figure 4I, 4J). In addition, our stratified analysis revealed that the m6A-scoring signature had a prognostic value that was independent of age, sex, grade, stage, and TNM staging (*p* < 0.05; Supplementary Figure 6). Therefore, the m6A-scoring signature is a reliable and independent prognostic biomarker for assessing the prognosis of HCC patients.

### The m6A-scoring signature characterized by distinct immunotherapy landscapes

**Figure 5:**
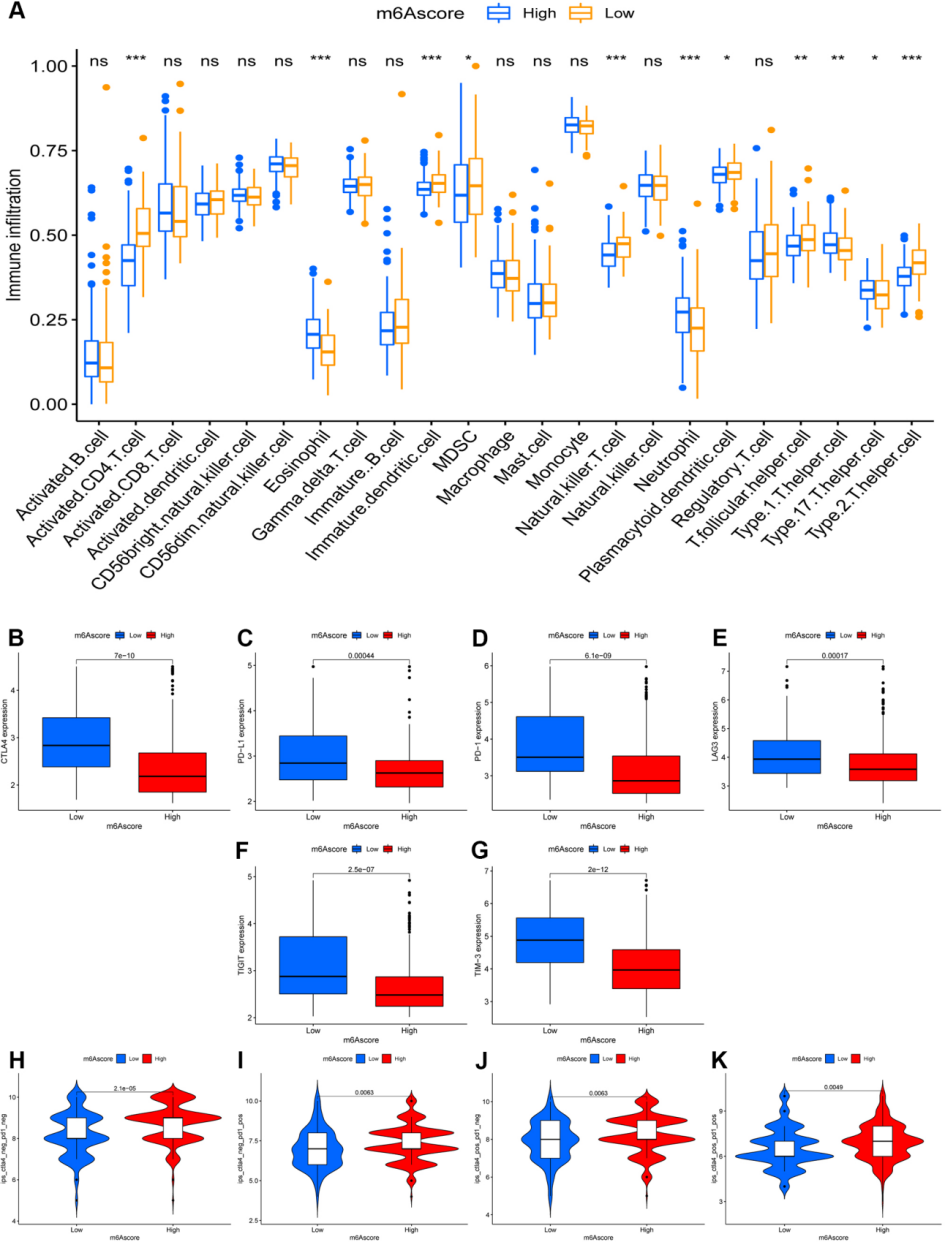
**The role of m6A-scoring signature in immunotherapy.** (**A**) Differences in immune cell infiltration of m6A-scoring signature (*, P < 0.05; **, P < 0.01; ***, P < 0.001; ns, no significant). (**B**–**G**) Expression of immune checkpoints among low and high m6A score groups. Immune checkpoints including CTLA4 (**B**), PD-L1 (**C**), PD-1 (**D**), LAG3 (**E**), TIGIT (**F**), TIM-3 (**G**). (**H**–**K**) The relative distribution of immunophenoscore (IPS) was also compared between low and high m6A score groups.

Unexpectedly, the m6A-scoring signature was strongly associated with multiple immune cell infiltrates. Compared with the low-m6A score group, in the high-m6A score group, the levels of eosinophils, neutrophils, Type 1 T helper cell, and Type 17 T helper cell infiltration were higher, whereas the levels of activated CD4 T cell, immature dendritic cell, MDSC, natural killer T cell, plasmacytoid dendritic cell, T follicular helper cell, and Type 2 T helper cell were lower (Figure 5A). In the testing dataset, patients with high m6A scores had higher levels of activated B cell, activated CD4 T cell, activated CD8 T cell, activated dendritic cell, immature B cell, monocytes, follicular helper cell, and Type 2 T helper cell, but a lower eosinophil count. In the past few years, immunotherapy against the immune checkpoints CTLA-4, PD-L1, and PD-1 has progressed well and has increasingly received attention. Therefore, we further investigated the differences in the expression of immune checkpoints between the two groups. We found significant differences in the expression of CTLA4, PD-L1, PD-1, LAG3, TIGIT, and TIM-3 between the two groups (Figure 5B–5G). In combination with the results of the m6A-scoring signature survival analysis, patients with a high m6A score showed a significant clinical advantage.

Considering the strong association between the m6A-scoring signature and immune response, the response to ICI treatment represented by CTLA-4/PD-1 inhibitors was further explored in terms of immunotherapy between the two groups. The results showed that patients in the high-m6A score group had higher ICI scores in the anti-PD-1 treatment alone (Figure 5I), anti-CTLA-4 treatment alone (Figure 5J), or in the combined anti-CTLA-4/PD-1 treatment cohort (Figure 5K), reflecting significant treatment and clinical benefits. Taken together, our findings strongly suggest that the m6A-scoring signature is associated with the response to immunotherapy.

## DISCUSSION

m6A methylation is the most prevalent form of mRNA modification and it plays a vital role in the regulation of gene expression at the post-transcriptional level [19]. Evidence that highlights the importance of abnormal m6A methylation in cancer progression via the regulation of many biological processes, including cell differentiation, immunoreaction, and miRNA editing is accumulating [20]. Moreover, the accumulated data suggest that m6A modification via m6A regulators is associated with inflammation, tumor microenvironment (TME), and immune response [21]. Exploration of m6A regulator-mediated methylation modification patterns and the characterization of TME infiltration may facilitate the identification of potential prognostic signatures and help determine the immunotherapeutic strategies for HCC [22].

In order to further clarify the clinical or biological value of these m6A regulators, we performed consensus clustering and divided the TCGA-LIHC cohort samples into subgroups based on the expression of 23 m6A regulators. Thus, we identified three different m6A methylation-modification patterns in LIHC based on 21 m6A regulators. Among these three patterns, the prognosis of the m6Acluster B subgroup was optimal, whereas the m6Acluster A had the worst prognosis. Interestingly, these three subgroups based on m6A regulator-expression patterns had different TME cell-infiltration characterization and biological behavior. The m6Acluster B was characterized by the activation of adaptive immunity, which corresponded to the immune-inflamed phenotype whereas m6Acluster A was characterized by the suppression of immunity, which corresponded to the immune-desert phenotype. The result of GSVA revealed that the m6Acluster A was mainly enriched in cell cycle and in RNA degradation whereas m6Acluster B was mainly enriched in the tumor metabolism-related pathways. The m6Acluster B of LIHC infiltrated by abundant immune cells in the TME was considered a hot tumor [23, 24], and this cluster of LIHC patients had a good immunotherapeutic effect and less drug resistance, which resulted in a better prognosis. Moreover, tumor metabolism in the m6Acluster B of LIHC could lead to the relative inhibition of tumor growth and good prognosis, which was also consistent with the data of our study [25]. The immune-desert phenotypes were linked to immune tolerance and ignorance, and the lack of T cells [26]. Interestingly, we found that the m6Acluster A exhibited significant RNA degradation and apoptosis that was regulated by the cell cycle [27, 28]. Therefore, it was unsurprising that the patients in the m6Acluster A subgroup had a poor prognosis.

Based on the abovementioned m6A modification patterns in HCC, we further explored m6A-related transcriptional expression patterns among these modification patterns and identified 5372 m6A phenotypic DEGs, which were referred to as the m6A-related signature genes. Interestingly, these genes mainly correlated with hepatitis virus infection and cell-cycle pathways, which are involved in the oncogenesis and progression of HCC [29–31]. Similar to the clustering results of the m6A modification phenotypes, three genomic subtypes (geneCluster A/B/C) were identified based on the m6A signature genes. Further analysis revealed that HCC patients in geneCluster B had the best prognosis whereas the HCC patients in geneCluster A had the worst prognosis, which reiterates that m6A modification has great significance in differentiating different types of HCC patients and, thus, in selecting different therapeutic strategies. Due to the individual heterogeneity of m6A modification, the above-described cluster could not quantify the m6A-modification patterns of individual tumors. Thus, we then constructed a scoring mechanism to calculate the m6A-modification pattern of individual HCC patients based on the m6A signature genes. Moreover, survival analysis revealed that the m6Ascore was an independent prognostic biomarker for HCC.

Another important finding of our study is that the m6A-scoring signature was significantly associated with immune cell infiltration and immune checkpoints. We found that high m6A-scoring was positively correlated with high levels of activated CD4+ T cell, natural killer T cell, and dendritic cell. Consistent with the results of previous studies which indicated that HCC treated with dendritic cell therapy was demonstrated to significantly improve the OS, HCC patients with high m6A-scoring had a better OS [32]. Previous studies revealed that the m6A scoring signature could guide immunotherapeutic strategies. Sheng et al. constructed an m6A-scoring signature in glioma that could predict therapeutic efficacy and patient prognosis [11]. Interestingly, another m6A scoring signature was associated with sorafenib treatment and anti-PD-1 immunotherapy response in HCC [33]. In our study, the m6Ascore was higher in HCC patients who had a good response to immunotherapy, including PD-1 and CTLA4, which could also account for the result that HCC patients with a high m6A-scoring had a better OS. Thus, we could evaluate the efficacy of adjuvant chemotherapy and the clinical response of HCC patients to anti-PD-1/PD-L1 immunotherapy by using the m6Ascore. However, whether the m6A scoring signature could distinguish the high-risk HCC cases who received immunotherapy is unclear and needs to be determined in further studies.

## CONCLUSIONS

This study clarified the extensive regulatory mechanisms of m6A methylation modification in the HCC microenvironment. Due to the heterogeneity of m6A modification patterns, we constructed a m6A-scoring signature to identify individual tumor m6A-modification patterns that could enhance our understanding of the TME cell-infiltrating characterization and guide the implementation of more effective immunotherapeutic strategies.

## Supplementary Material

Supplementary Figures
