# Supplementary material for: m6A regulator-mediated methylation modification patterns and tumor microenvironment infiltration characterization in hepatocellular carcinoma
Source: Aging (Albany NY). 2021 Aug 30;13(16):20698–715. doi: 10.18632/aging.203456 (PMC8436903; doi:10.18632/aging.203456)
Supplement: Supplementary Figures [file aging-13-203456-s001.pdf]

SUPPLEMENTARY FIGURES

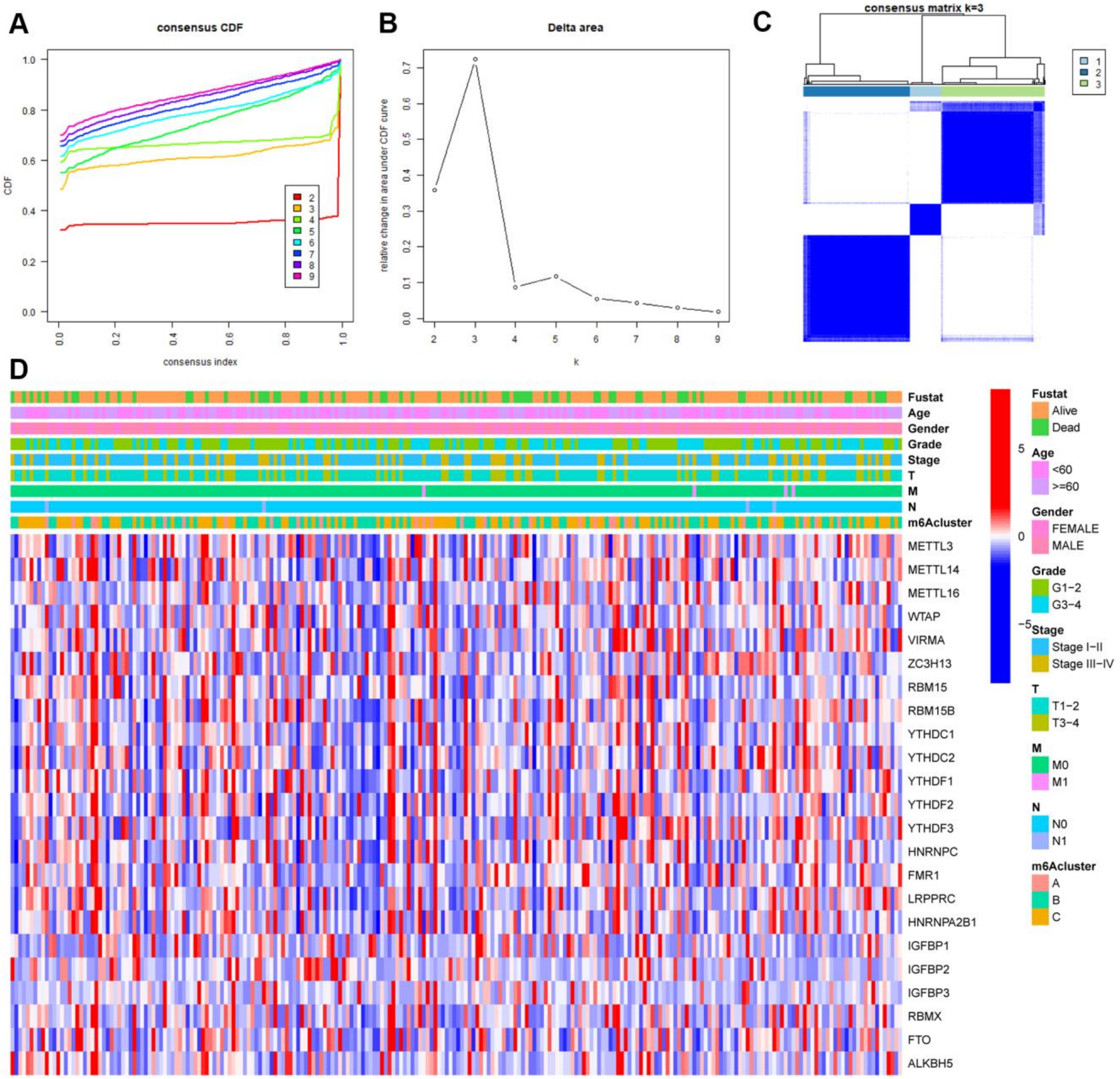

Supplementary Figure 1. Consensus clustering of m6A regulators. (**A**) Consensus among clusters with number k of each category. (**B**) Delta area curves for consistent clustering, representing the relative change in area under the cumulative distribution function (CDF) curve for each category number k versus k-1. The horizontal axis represents the number of categories k and the vertical axis represents the relative change in area under the CDF curve. (**C**) The consistency clustering was applied to obtain a colored heat map of the consistency matrix corresponding to k=3. The color gradient indicates the consistency values between 0 and 1, with white representing 0 and dark blue representing 1. (**D**) A heatmap annotated with survival status (Fustat), age, gender, Stage, Grade, TNM staging and m6A methylation modification patterns (m6Acluster).

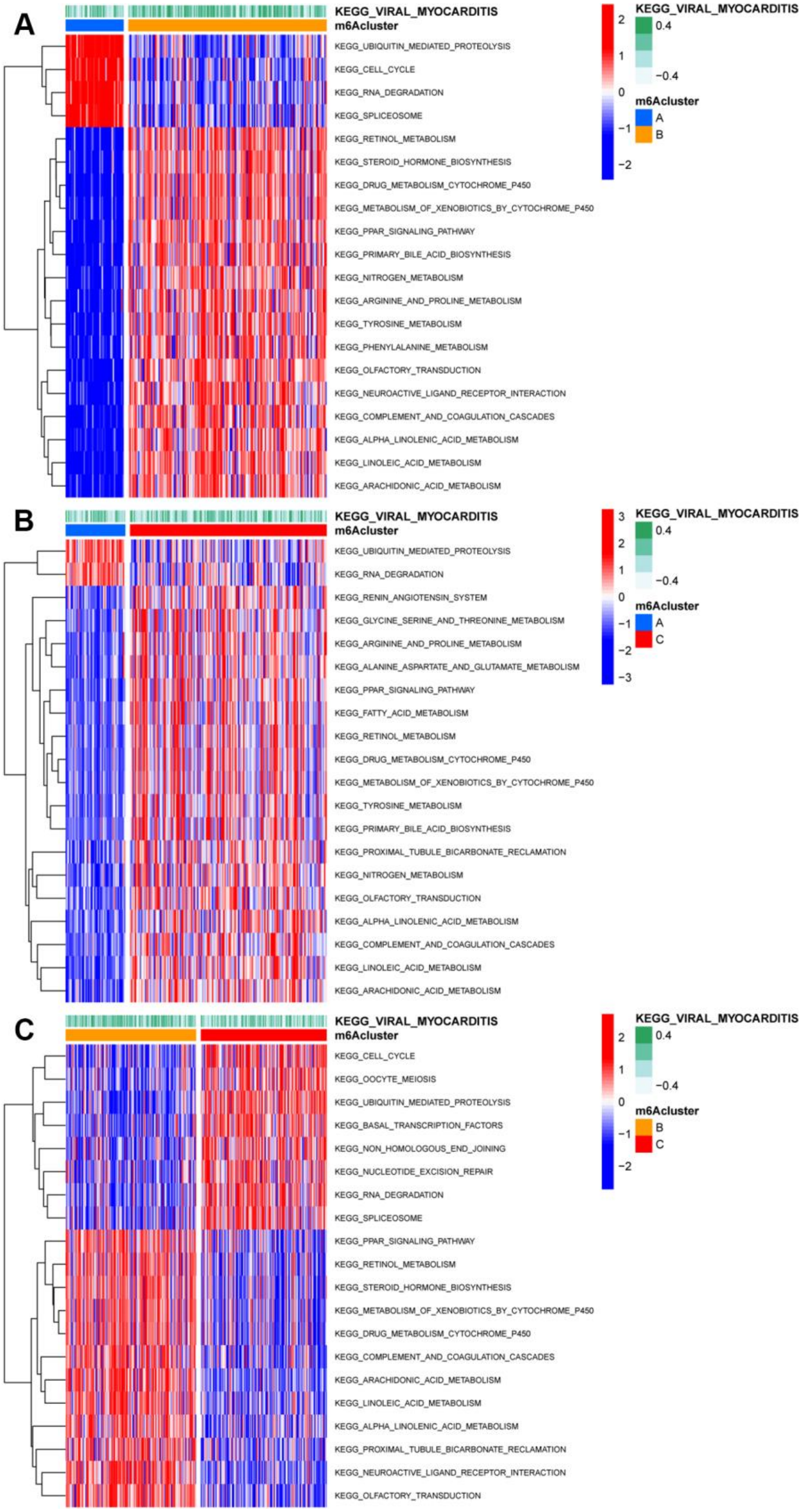

Supplementary Figure 2. Biological pathway of m6A methylation modification pattern. In the heatmap, red represents the activated pathway and blue represents the inhibited pathway. (**A**) m6Acluster A and m6Acluster B. (**B**) m6Acluster A and m6Acluster C. (**C**) m6Acluster B and m6Acluster C.

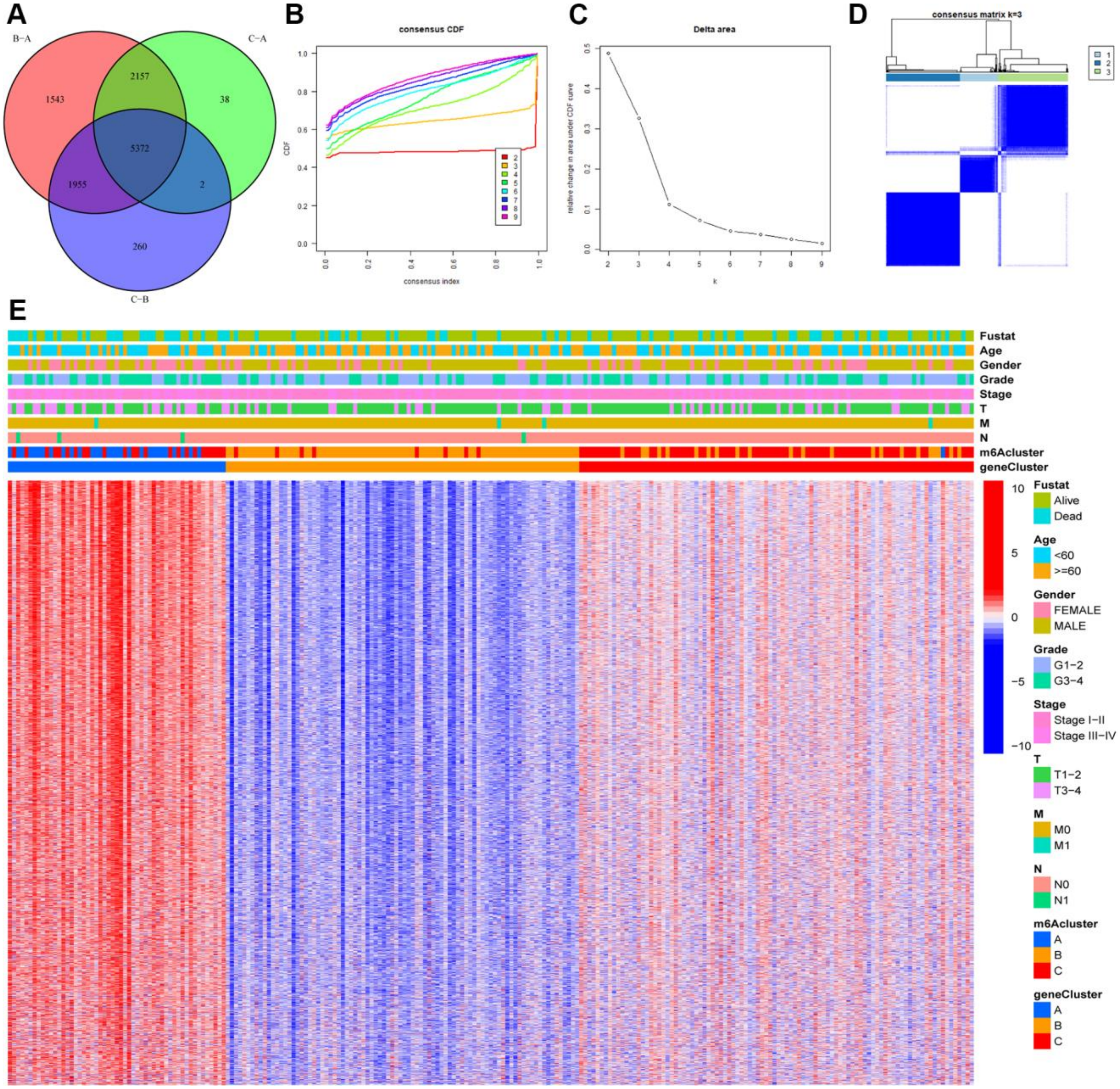

Supplementary Figure 3. Consensus clustering of differentially expressed genes (DEGs) for the m6A modification patterns. (**A**) 5372 m6A modification patterns related genes shown in Venn diagram. (**B**) Consensus among clusters with number k of each category. (**C**) Delta area curves for consistent clustering, representing the relative change in area under the cumulative distribution function (CDF) curve for each category number k versus k-1. The horizontal axis represents the number of categories k and the vertical axis represents the relative change in area under the CDF curve. (**D**) The consistency clustering was applied to obtain a colored heat map of the consistency matrix corresponding to k=3. The color gradient indicates the consistency values between 0 and 1, with white representing 0 and dark blue representing 1. (**E**) A heatmap annotated with survival status (Fustat), age, gender, Stage, Grade, TNM staging, m6A methylation modification patterns (m6Acluster) and m6A modification genomic phenotypes (geneCluster).

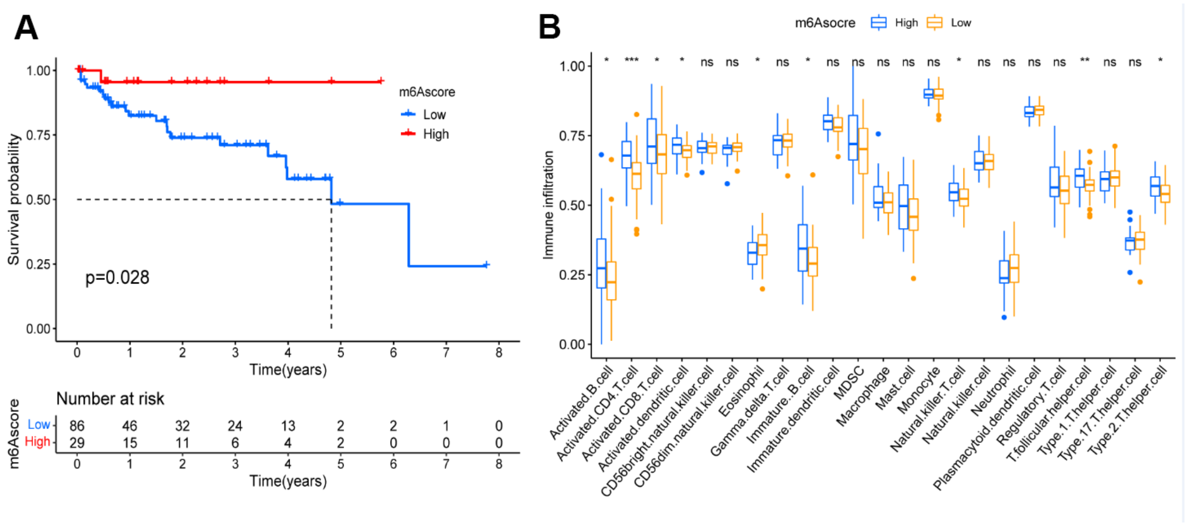

Supplementary Figure 4. Validation of the m6A-scoring signature in the GSE76427 cohort. (**A**) The overall survival of m6A-scoring signature using Kaplan–Meier in Log-rank test. (**B**) Differences in immune cell infiltration of m6A-scoring signature (*, P < 0.05; **, P < 0.01; ***, P < 0.001; ns, no significant).

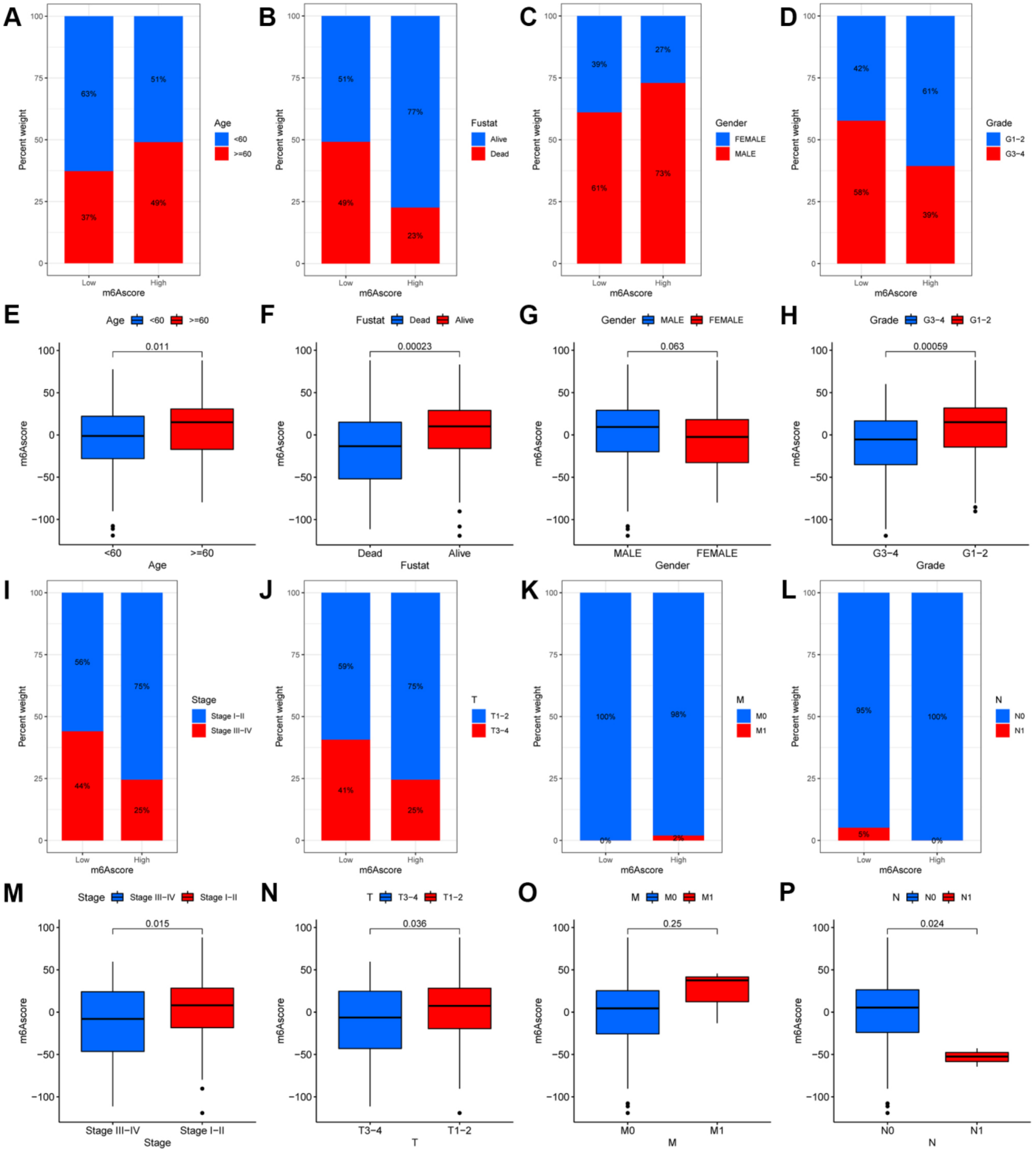

Supplementary Figure 5. Clinical correlation analysis of the m6A-scoring signature with distinct clinical subgroups. The percent weight of patients with distinct clinical subgroups in low or high m6A score groups (including patients with Age (**A**), survival status (Fustat) (**B**), Gender (**C**), Grade (**D**), Stage (**I**), T staging (**J**), M staging (**K**), N staging (**L**)). Differences in m6A-scoring signature among Age (**E**), Fustat (**F**), Gender (**G**), Grade (**H**), Stage (**M**), T staging (**N**), M staging (**O**), N staging (**P**), of which Age, Fustat, Grade, Stage, T staging, N staging were statistically significant.

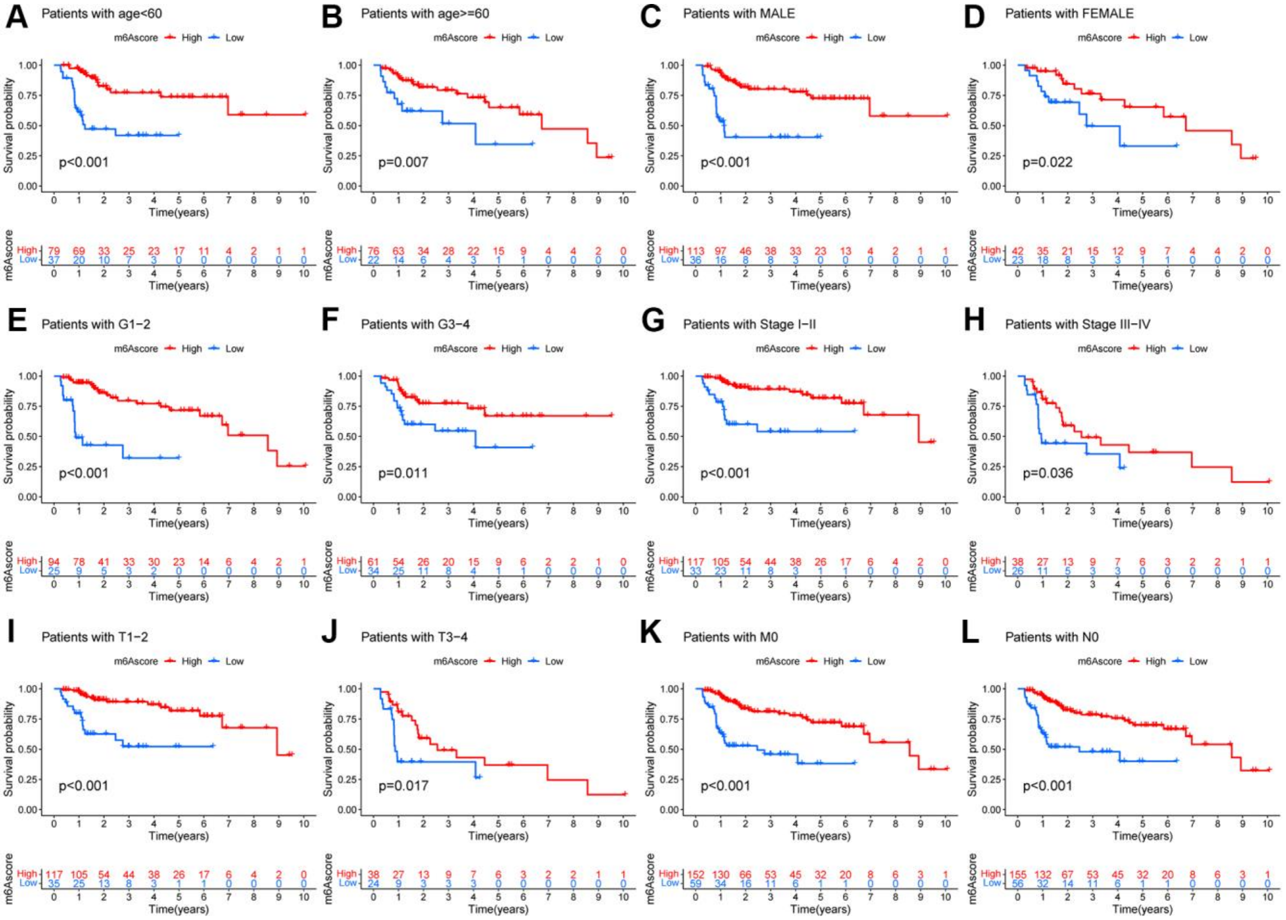

Supplementary Figure 6. The prognostic value of the m6A-scoring signature with distinct clinical subgroups using Kaplan–Meier in Log-rank test. The patients with Age<60 (**A**), Age >60 (**B**), Male (**C**), Female (**D**), G1-2 (**E**), G3-4 (**F**), Stage I+II (**G**), Stage III+IV (**H**), T1+2 (**I**), T3+4 (**J**), M0 (**K**), N0 (**L**) were statistically significant, and the survival of patients in the high m6A score group is better than that in the low m6A score group.
